# Supplementary material for: Empirical methods for controlling false positives and estimating confidence in ChIP-Seq peaks
Source: BMC Bioinformatics. 2008 Dec 5;9:523. doi: 10.1186/1471-2105-9-523 (PMC2628906; doi:10.1186/1471-2105-9-523)
Supplement: Additional File 1 — A variety of html documents from the USeq web site detailing the available applications, their best usage, output file type descriptions, command line menus, etc. [file 1471-2105-9-523-S1.zip › USeqUserGuides/JavaDoc.zip!/JavaDoc/trans/main/RandomizeLabels.html]

RandomizeLabels


|  |  |  |
| --- | --- | --- |
| **Overview** \| **Package** \| **Class** \| **Use** \| **Tree** \| **Deprecated** \| **Index** \| **Help** | |  |
| **PREV CLASS**   **NEXT CLASS** | **FRAMES**    **NO FRAMES**     **All Classes** |  |
| SUMMARY: NESTED \| FIELD \| CONSTR \| METHOD | DETAIL: FIELD \| CONSTR \| METHOD |  |


---


## trans.main Class RandomizeLabels

```
java.lang.Object
  trans.main.RandomizeLabels
```

---

``` public class RandomizeLabels extends java.lang.Object ```

---

**Constructor Summary**

`RandomizeLabels(float[][] t,
float[][] c)`


| **Method Summary** | |
| --- | --- |
| `java.lang.String` | `concatinate()`             Concat name numbers to a String. |
| `float[][][]` | `fetchPermutatedArrays()`             Attempts to fetch a unique, unseen permutation of the t[][] and control[][] arrays. |
| `java.lang.String` | `groupName(trans.main.RandomizeLabels.ArrayName[] ans)`             Returns a sorted groupName where the sort is on the treatments and controls, not both. |
| `static void` | `main(java.lang.String[] args)` |
| `void` | `makeArrayNames(float[][] t, float[][] c)`             Convert intensity arrays into an ArrayName[]. |
| `void` | `permutate()`             Randomly permutates the ArrayName[]. |
| `trans.main.RandomizeLabels.ArrayName[]` | `randomize(trans.main.RandomizeLabels.ArrayName[] ans)` |

**Methods inherited from class java.lang.Object**

`equals, getClass, hashCode, notify, notifyAll, toString, wait, wait, wait`

**Constructor Detail**

### RandomizeLabels

```
public RandomizeLabels(float[][] t,
                       float[][] c)
```


**Method Detail**

### main

```
public static void main(java.lang.String[] args)
```

---


### fetchPermutatedArrays

```
public float[][][] fetchPermutatedArrays()
```

:   Attempts to fetch a unique, unseen permutation of the t[][] and control[][] arrays.
    Returns null if no more unique permutations are possible.
    Otherwise returns a float[2][][] of treatment then control arrays.

---


### randomize

```
public trans.main.RandomizeLabels.ArrayName[] randomize(trans.main.RandomizeLabels.ArrayName[] ans)
```

---


### groupName

```
public java.lang.String groupName(trans.main.RandomizeLabels.ArrayName[] ans)
```

:   Returns a sorted groupName where the sort is on the treatments and controls, not both.

---


### permutate

```
public void permutate()
```

:   Randomly permutates the ArrayName[].

---


### concatinate

```
public java.lang.String concatinate()
```

:   Concat name numbers to a String.

---


### makeArrayNames

```
public void makeArrayNames(float[][] t,
                           float[][] c)
```

:   Convert intensity arrays into an ArrayName[].


---


|  |  |  |
| --- | --- | --- |
| **Overview** \| **Package** \| **Class** \| **Use** \| **Tree** \| **Deprecated** \| **Index** \| **Help** | |  |
| **PREV CLASS**   **NEXT CLASS** | **FRAMES**    **NO FRAMES**     **All Classes** |  |
| SUMMARY: NESTED \| FIELD \| CONSTR \| METHOD | DETAIL: FIELD \| CONSTR \| METHOD |  |


---
